# Sharing, synthesis and sustainability of data analysis for epidemic preparedness in Europe

**DOI:** 10.1016/j.lanepe.2021.100215

**Published:** 2021-10-07

**Authors:** Adam J Kucharski, Emma B Hodcroft, Moritz U G Kraemer

**Affiliations:** a Centre for Epidemic Preparedness and Response, London School of Hygiene & Tropical Medicine, UK; b Centre for Mathematical Modelling of Infectious Diseases, London School of Hygiene & Tropical Medicine, UK; c Institute of Social and Preventive Medicine, University of Bern, Switzerland; d Swiss Institute of Bioinformatics, Lausanne, Switzerland; e Department of Zoology, University of Oxford, UK

When it came to COVID-19 in Europe, early 2021 echoed early 2020. Each year started in the shadow of a new infectious disease threat, with SARS-CoV-2 spreading widely in 2020, followed by the Alpha and Beta variants in 2021. These viruses initially moved undetected between countries, before causing large local outbreaks and triggering the implementation of strict control measures. However, there were also some crucial differences in 2021: researchers had a better understanding of the infection, effective vaccines were starting to roll-out, and health agencies had more real-time data streams available. In terms of tracking and responding to epidemics, the COVID-19 pandemic has highlighted some promising developments in data integration and analysis, but it has also illustrated some key challenges. Novel data streams have the potential to reshape epidemic preparedness and response, but there remains a pressing need to address issues around sharing, sustainability and scalability that could hinder future epidemic preparedness.

Challenges around data analysis span the whole life cycle of an epidemic. Early on, there is a need for fundamental insights into the epidemiological characteristics of a novel infection, from transmission potential to natural history. This requires a rapid scale-up of testing and sequencing, a fast assessment of clinical impact, and open sharing of early findings. As outbreaks grow, there is a need for forecasting of disease dynamics, estimation of potential burden and evaluation of interventions. Coordination and equitable data generation is critical here, to ensure that recommendations and responses are relevant to all affected demographics and settings. In the later stages, attention turns to estimation of vaccine effectiveness and tracking flare-ups and evolutionary dynamics. This can include analysis of longer-term datasets including cohort studies, which can provide insights into epidemic processes over months or even years.

## 1 Shareability and standardisation

Effective epidemic response hinges on fast and reliable data sharing, particularly given the global nature of infectious disease threats. From tracking COVID-19 variants to analysis of measles flare-ups and influenza vaccine strain selection, efficient and secure data sharing enables responses to be based on the best possible evidence. It can also support rapid follow-up analysis that builds on earlier work, reducing repetition. However, there can be challenges in ensuring rapid analysis and sharing while maintaining appropriate credit and quality control [1]. Community-based platforms for data sharing and analysis, such as the COVID-19 forecast hubs co-ordinated by CDC and ECDC [2,3] or the global.health line list repository supported by multiple institutions [4], provide one route to balancing these considerations. Such initiatives can also be integrated with novel data collection, from arbovirus distributions [5] to population immunological landscapes [6]. Crucially, reward structures will need to evolve to value real-time data generation and processing that can unlock valuable epidemic insights [7]. This could include dedicated funding streams, sustainable career paths and stronger open science mandates for teams working on policy-relevant data projects.

In addition to sharing of specific data outputs, there are major benefits to enabling linkage between disparate data streams, particularly combinations that provide orthogonal information about the epidemic. For example, with each new SARS-CoV-2 variant that has emerged, scientists and researchers across multiple specialties have been able to generate unprecedented data about its potential impact. Clinical research, epidemiological analysis, mathematical modelling, phylogenetics, and studies looking at potential changes in antibody and ACE2 binding can provide detailed, multi-faceted insights [8]. However, there remain obstacles to both collating such data and sharing them in an ethical way; detailed analysis can often involve potentially sensitive personal information, such as linking one or more of pathogen genetic sequences, demographic metadata, human genomics, clinical outcomes and data from wearable devices [9].

To protect privacy around sensitive clinical data, platforms such as OpenSAFELY [10] have developed pipelines allowing researchers to pose questions to electronic health record datasets without viewing the underlying raw data. Meanwhile, projects like Google Community Mobility Reports and Facebook Disease Prevention Maps have generated privacy-preserving data products around mobility and behaviour that can be more widely used by disease researchers [11]. As routine use of large-scale epidemic datasets increases, there will be a need for clear international ethical standards, particularly when information traverses borders or public-private partnerships. Such efforts could draw on existing work by organisations such as the Ada Lovelace Institute and Open Data Institute, with coordination provided by relevant regional and international bodies. In the development of digital COVID-19 vaccination certification, for example, the European Commission has overseen standards within the continent, but integration with wider countries remains a crucial challenge.

More broadly, countries need to proactively plan for how to balance legal aspects of privacy and public health during epidemics. For example, concerns relating to the General Data Protection Regulation (GDPR) in Denmark meant that no SARS-CoV-2 sequences could be shared internationally for almost two months in early 2021, until a new law was passed [12]. In contrast, South Korea amended certain privacy laws after its 2015 MERS outbreak to accelerate data sharing in the event of a future infectious disease emergency [13]. To build public trust, such initiatives will need to be supported by transparent communication by governments and health agencies. It would also be beneficial to incorporate issues relating to privacy and health into routine education programmes.

Even if linked data can be shared and analysed within a country, there can also be limitations to integration across geographies and populations. For example, mobility datasets may not pass across country boundaries [11], serological studies and assays may not be standardised across labs and territories [14], and sample selection for sequencing may vary between countries [7]. These issues can be exacerbated by regional inequities in data generation, from testing capacity to smartphone coverage, leading to systematic biases in common datasets. As a first step, ensuring that existing national and international data portals provide ways to record why and how data is collected, and any known biases, would mean that subsequent analyses can try to control for these effects [15]. Longer term, making sampling, standardization, and sharing strategies transparent will allow better integration of available information. In real-time, data generation efforts will often need to be adaptive and decentralised, but data science capacity also needs to be in place within health agencies to ensure cross-scale interoperability. Moreover, enabling more equitable data access and generation – ideally through sustainable long-term investments to develop local resources, rather than externally-led project-specific funding – will reduce uneven coverage and improve the reliability and generalisability of resulting insights.

## 2 Sustainability and scalability

As well as being shareable, data analysis also needs to be sustainable. The volume and diversity of data that has been generated during the COVID-19 pandemic has been unparalleled. To process and analyse these datasets, scientists have built new tools and found ways to improvise and upgrade existing software to handle large, previously imaginable data inputs. The urgency of the pandemic has driven a proliferation of creation and improvement, but generally few of these tools and resources have long-term funding, making sustainability difficult. For example, several key resources – such as the COVID Tracking Project in the US [16] – were created from scratch by committed, but often short-term, volunteers. In contrast, successful open source data and software need ongoing maintenance to rapidly respond to the changing needs of their communities of users. This is far from a new problem: the cycle of redeveloping tools to replace those that are no longer maintained, often linked to precarious academic positions and lack of funding, was documented long before 2020 [17]. However, the COVID-19 pandemic has put into sharp focus the need to take a longer view of resource development and maintenance.

In future, integrated programmes linking partners including academia, government departments, research funders, health organisations, and private sector groups could enable efficient coordination of analysis development and clear responsibility for maintenance and implementation. These could build on existing structures such as WHO collaborating centres and the aforementioned forecast hubs; these structures provide academic groups with access to data and partnerships, while giving governments and health agencies access to scientific insights. Such mechanisms would also ensure decision makers have access to robust consensus estimates from multiple research teams with an established track record, rather than relying on ad hoc analysis from reactively convened groups. Defining clear responsibility for upstream tasks such as data generation and methodological development as well as downstream implementation and communication would help ensure that key tasks are not neglected during an epidemic. This would mean countries are better placed not only for the next pandemic but also for the overall growth in demand for real-time epidemic analyses that data advances are bringing.

Countries will also need to look at data capacity beyond their own borders, rather than waiting until a threat has arrived domestically to gain vital insights. The COVID-19 pandemic has been a sharp reminder of how interconnected we are. What threatens some of us can soon threaten all of us, from the initial emergence and spread of SARS-CoV-2, to the recent variants of concern that spread worldwide despite severe restrictions on global travel. Alongside the spread of infection, we have seen the spread of information; societies have rarely benefited so clearly and immediately from information shared globally. From evaluating the effectiveness of interventions to tracking pathogen evolution, there is huge value in the accrual of information from across the globe. This also means having capacity in place to generate these data in the first place. While many countries have had dramatically scaled up their public health and data activities in 2020/21, some have been able to draw on resources built for analysis of other infectious diseases; the Democratic Republic of Congo and South Africa adapted infrastructure developed for Ebola and HIV to generate some of the first and the most sequences in Africa, respectively [18,19].

Even so, data streams such as diagnostics and sequencing remain hugely disparate. As of mid-May 2021, for every 26 SARS-CoV-2 sequences the United Kingdom had generated, the entire continent of Africa had produced only 1. It is very much in wealthy countries’ interests to ensure global surveillance capacities are consolidated and expanded. Inequities not only translate to local areas being unable to access situational awareness in the way some rich countries have, it also means a biased and incomplete global picture of the COVID-19 pandemic and a reduced ability to detect emerging variants of concern. There are also inequities in how data are used, with SARS-CoV-2 variants identified in LMICs being considered for candidate components in updated vaccines that in turn will not be easily accessible to those LMICs. Addressing these disparities will be essential if the world is to avoid ongoing major COVID-19 epidemics in 2022 that have echoes of 2020 and 2021.

Synthesis of multiple data streams in a shareable, sustainable way will also improve countries’ ability to respond to non-COVID-19 threats. Large scale public health measures during 2020/21 have disrupted the dynamics of seasonal infections such as influenza and respiratory syncytial virus in the southern hemisphere, leading to large out-of-sync epidemics [20], and Europe is likely to face similar atypical resurgences in late 2021, as well as the ongoing threat of novel emerging infections and known risks such as antimicrobial resistance. COVID-19 has revealed weaknesses in how countries collect, analyse and act upon data, but it also provided innovations, insights and collaborations that could transform how countries prepare and respond to epidemics in future.

## Funding

AJK is supported by a Sir Henry Dale Fellowship jointly funded by the Wellcome Trust and the Royal Society (grant Number 206250/Z/17/Z). MUGK is supported by a Branco Weiss Fellowship. EBH is supported by the Swiss National Science Foundation (SNSF) (grant number 31CA30_196046).

## Author contributions

AJK, EBH and MUGK developed and wrote the manuscript.

## Declaration of Interests

EBH reports personal fees from GLG, personal fees from Coleman Research, outside the submitted work. AJK and MUGK have no interests to disclose.
